# Using Nonexpert Online Reports to Enhance Expert Knowledge About Causes of Death in Dental Offices Reported in Scientific Publications: Qualitative and Quantitative Content Analysis and Search Engine Analysis

**DOI:** 10.2196/15304

**Published:** 2020-04-17

**Authors:** Meike Gaiser, Joachim Kirsch, Till Sebastian Mutzbauer

**Affiliations:** 1 Institute for Anatomy and Cell Biology University of Heidelberg Heidelberg Germany; 2 Maxillofacial Surgery and Dental Anesthesiology Mutzbauer & Partner Zürich Switzerland

**Keywords:** dental death, dental practice, dental sedation, risk, internet search engine

## Abstract

**Background:**

Fatalities rarely occur in dental offices. Implications for clinicians may be deduced from scientific publications and internet reports about deaths in dental offices.

**Objective:**

Data involving deaths in dental facilities were analyzed using Google as well as the PubMed database. By comparing both sources, we examined how internet data may enhance knowledge about deaths in dental offices obtained from scientific medical publications, which causes of death are published online, and how associated life-threatening emergencies may be prevented.

**Methods:**

To retrieve relevant information, we searched Google for country-specific incidents of death in dental practices using the following keywords: “death at the dentist,” “death in dental practice,” and “dying at the dentist.” For PubMed searches, the following keywords were used: “dentistry and mortality,” “death and dental treatment,” “dentistry and fatal outcome,” and “death and dentistry.” Deaths associated with dental treatment in a dental facility, attributable causes of death, and documented ages of the deceased were included in our analysis. Deaths occurring in maxillofacial surgery or pre-existing diseases involved in the death (eg, cancer and abscesses) were excluded. A total of 128 cases from online publications and 71 cases from PubMed publications that met the inclusion criteria were analyzed using chi-square statistics after exclusion of duplicates.

**Results:**

The comparison between the fatalities from internet (n=117) and PubMed (n=71) publications revealed that more casualties affecting minors appeared online than in PubMed literature (online 68/117, 58.1%; PubMed 20/71, 28%; *P*<.001). In PubMed articles, 10 fatalities in patients older than 70 years of age were described, while online sources published 5 fatalities (*P*=.02). Most deaths, both from internet publications and PubMed literature, could be assigned to the category *anesthesia, medication, or sedation* (online 80/117, 68.4%; PubMed 25/71, 35%; *P*<.001). Deaths assigned to the categories *infection* and *cardiovascular system* appeared more often in the PubMed literature (*infection*: online 10/117, 8.5%; PubMed 15/71, 21%; *P*=.01; *cardiovascular system*: online 5/117, 4.3%; PubMed 15/71, 21%; *P*<.001). Furthermore, sedative drugs were involved in a larger proportion of fatal incidents listed online compared to in PubMed (online 41/117, 35.0%; PubMed: 14/71, 20%, *P*=.03). In the United States, more deaths occurred under sedation (44/96, 46%) compared to those in the other countries (Germany and Austria 1/17, 6%, *P*=.002; United Kingdom 1/14, 7%, *P*=.006).

**Conclusions:**

Online and PubMed databases may increase awareness of life-threatening risks for patients during dental treatment. Negative aspects of anesthesia and sedation, as well as the number of deaths of young patients, were underestimated when reviewing PubMed literature only. Medical history of patients, medication dosages, and vital function monitoring are significant issues for practitioners. A high-impact finding from online reports was the underestimation of risks when performing sedation and even general anesthesia. Detailed knowledge of the definition and understanding of *deep sedation* and *general anesthesia* by dentists is of major concern. By avoiding potentially hazardous procedures, such as sedation-aided treatments performed solely by dentists, the risk of treatment-induced life-threatening emergencies may be reduced.

## Introduction

Fortunately, complications with a fatal outcome rarely occur in dental offices. In the United Kingdom, for example, one dental-related death occurs per 464-758 dentist years [1]. Fatalities may occur not only in elderly and multimorbid patients but also in younger patients, especially in connection to more extensive surgical procedures [2]. The probability that one will experience a medical emergency in a dental setting is remarkably higher; in the United Kingdom, 9 to 11 emergencies occur over a period of 40 years as a dentist [1]. In Saxony, Germany, a study was conducted to find out how often medical emergencies occur in the practice of dentistry; more than 50% of the respondents reported 1 to 3 medical emergencies having occurred within a year and 36% reported the occurrence of up to 10 emergencies [3]. However, it is not possible to determine the fatality risk of life-threatening emergencies happening in dental facilities.

Furthermore, it cannot be determined if fatalities occurred coincidentally or if the dentists’ treatments contributed to the fatal events. Despite the low incidence of fatalities in dental facilities, it is important to analyze the causes of death in order to gain knowledge about how these life-threatening emergencies could be prevented. Publications in scientific medical journals may contribute to awareness about medical emergencies occurring in dental facilities. However, it is assumed that additional information could be extracted from internet reports, which could enhance the knowledge about the background and pathogenesis of fatal events. Additional management strategies could be developed when considering information being published on the internet about medical emergencies having resulted in deaths. At best, dentists would avoid pursuing a life-threatening course of treatment, for example, by not performing interventions defined as at-risk procedures.

In 2017, Reuter et al [4] performed a systematic review examining deaths that were related to dental procedures. They reviewed various specialized literature libraries without date restrictions and extracted data from North America, Europe, Asia, and South America about causes and affected patients. A total of 148 fatalities, with an average patient age of 34.6 years, were investigated. In scientific medical literature, as well as on the internet, deaths in dental facilities associated with sedation or general anesthesia have been reported; however, other causes have also been reported [4-32].

The Google Trends database can display the search volume of a keyword, for instance, “dental.” Some differences in search volumes can be found for the keywords “dental” and “death” when comparing searches of these words in relation to the total counts of global searches using this database in the United States, the United Kingdom, and in German-speaking countries. However, because of a lack of data, probably due to a low number of searches, there are no statistics available regarding the search term “death dental office” in these respective countries. The term “dental” is searched for twice as often as the term “death” in the United States, the United Kingdom, and in Switzerland; in Germany and Austria, the term “death” is searched for even more seldomly in relation to the term “dental” (see Multimedia Appendix 1).

The importance of the internet in daily life is increasing. Patients use the internet not only to obtain information but also to exchange experiences and to rate doctors. In a study from 2017, it was demonstrated that the number of doctor ratings were increasing [5]. For clinicians, the internet offers additional resources and scientific insights into problems and trends that might be helpful in their daily work as well as for the prevention of diseases. Forums, for example, may deal with patient fears and could be helpful for clinicians as well; they may benefit from patients’ and laypersons’ points of view and adjust their treatments accordingly.

To the best of our knowledge, there is no overview of deaths in dental offices that has been published on the internet. Hence, we performed a qualitative and quantitative analysis of online publications and medical literature covering fatalities in dental offices in German-speaking countries, France, the United Kingdom, and the United States. In addition to the causes of death, patient groups were analyzed.

This study aims to examine content regarding death in dental treatment facilities that has been published on the internet and retrieved by the Google search engine and compare these findings with content extracted from the scientific medical literature.

The specific research topics of the study are as follows:

1. Which causes of death in dental offices are published on the internet?
2. How can the internet as a database enhance knowledge from scientific medical publications regarding deaths in dental offices?
3. What implications for clinicians can be deduced from internet reports and scientific publications about the reasons for deaths in dental offices?

## Methods

### Searches Using the Google Search Engine

Between December 21, 2016, and May 3, 2017, we performed a country-specific Google search for deaths in dental facilities. For the German language, where we used the URL top-level domains .de, .at, and .ch, German equivalents of the keywords “death at the dentist,” “death in dental practice,” and “dying at the dentist” were used. The terms “death at dentist,” “death in dental office,” and “dying at the dentist” were used for the English language, where the URL top-level domains .uk and .com were used. The term “death at dentist” was searched in the French language, where the URL top-level domain .fr was used.

An article related to a fatality was included when it had not been published in a medical specialist journal. Additionally, internet pages containing a collection of deaths in a dentist´s or a physician´s office were included. The internet was additionally searched for related content on May 31, 2018, and on October 31, 2018.

### Selection of Websites

Articles were included if a patient´s death had been associated with a dental treatment in a dental facility. Furthermore, the age of the casualty and the cause of death had to be indicated, as well as information about the type of anesthesia if the fatality was anesthesia related. In case of a severe pre-existing disease related to the oral cavity (eg, existing abscesses, infections, or tumor diseases), the case was excluded. Deaths related to oral and maxillofacial surgery were also excluded.

### Systematization of Articles Found Using Google

The systematization regarding the use of anesthesia was done as follows. Anesthesia was assigned to the category *general anesthesia* when “general anesthesia” or an intubation had been mentioned or when only the term “anesthesia” had been used by the author, even if local anesthesia had been used additionally. The category *sedation* was chosen when the terms “sedation,” “sedating drugs,” “sedate,” “tranquilization,” or “nitrous oxide” had been used.

Where there was a lack of information regarding anesthesia, an article only qualified for inclusion if the type of anesthesia had had no influence on the death or the information was unnecessary for the allocation of the cause of death. Cases with general anesthesia or sedation were evaluated with relation to the presence of an anesthesiologist. Furthermore, the anesthesia used was examined with relation to the administration of nitrous oxide. Table 1 lists the categories of the causes of death.

The patients were divided into the following age groups: 0-5 years, 6-17 years, 18-30 years, 31-55 years, 56-70 years, and >70 years. Imprecise age information was rounded; for instance, *early 30s* was rounded to 32 and *mid-70s* to 75.

**Table 1:** Categories of causes of death.

Categories	Examples
Anesthesia, medication, or sedation	Overdose, anaphylaxis, hypoxia, interactions, or any other recognizable cause
Airway or respiratory system	Suffocation, aspiration, false intubation, or laryngeal edema
Bleeding or coagulation	High blood loss, bleeding, or disseminated intravascular coagulation
Cardiovascular system	Heart attack, cardiac arrest, aneurysm, or air embolism
Infection	Bacterial, viral, or fungal infection; endocarditis, pneumonia, fasciitis, or mucormycosis
Others	Causative, but not temporary; temporary, but not causative

### Searches in PubMed

On May 11, 2017, a systematic literature search was performed in PubMed. We searched for the following Medical Subject Headings keywords: Dentistry AND Mortality OR Death AND Dental Treatment OR Dentistry AND Fatal Outcome OR Death AND Dentistry. Through filters, the search was limited to a period of the last 30 years and the term “humans.” PubMed was additionally searched on May 31, 2018, and on October 31, 2018.

### Selection and Systematization of Relevant Articles From PubMed

The inclusion criteria described in the above sections applied to online literature as well. Editorials, letters, and duplicates were excluded. Articles from Europe, North America, and Australia were included. The systematization was completed analogously to the online database.

### Comparison of Selected Publications by Chi-Square Statistics

Eventually, all included deaths from online publications were summarized and compared to the included deaths found in PubMed publications. After carefully reviewing all the cases, duplicates or possible duplicates were excluded before the evaluation of the results using chi-square statistics; the statistical software XLSTAT, version 2018 (Addinsoft), was used for analysis. For further statistical analysis and to definitively exclude duplicate cases, the deaths were assessed by year of occurrence and additionally divided into two groups: cases that occurred before 1995 and cases that occurred after 1994.

## Results

### Online Publications

#### Online Results in Total

**Figure 1:**
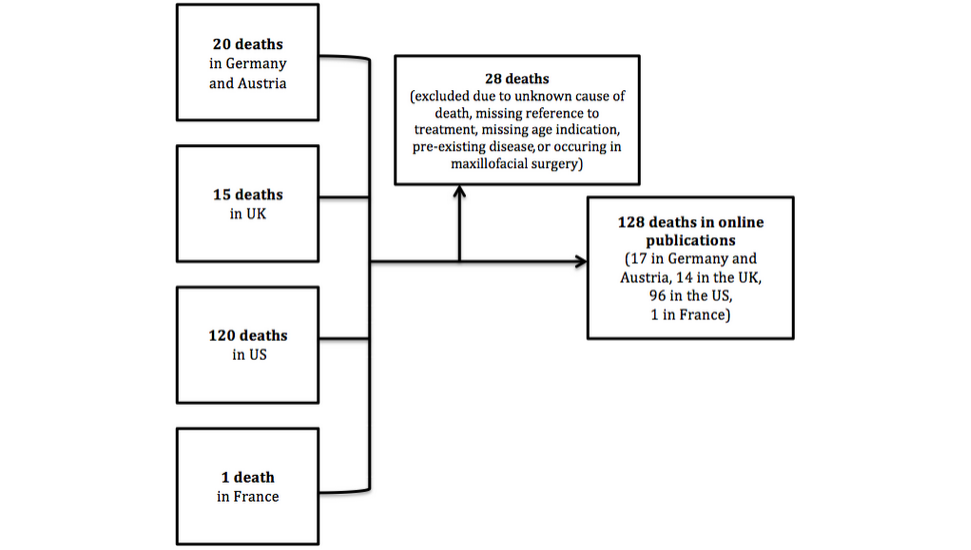
Flowchart for article selection from Google results on the basis of article title. UK: United Kingdom; US: United States.

A total of 18 casualties were found in Germany and 2 were found in Austria. Groups of deaths in the two countries were summarized as deaths in German-speaking countries. In Switzerland, no deaths were reported on the internet. A total of 120 deaths were reported from the United States, 15 from the United Kingdom, and 1 from France. After excluding 28 cases, there were a total of 128 casualties reported from online publications (see Figure 1).

#### Online Results for Germany and Austria

A total of 3 casualties were excluded from the 20 found in Germany and Austria. The mean age of the 17 remaining casualties was 19.3 years (SD 21.9; median 10 years). The dominating age group was 0-5 years and included 8 fatalities (8/17, 47%). A total of 2 of the deceased (2/17, 12%) were in the 6-17-year age group, whereas the age groups 18-30 years and 31-55 years had 3 patients in each (3/17, 18%). Of the 17 deceased, 1 (6%) was older than 70 years of age and the age group 56-70 years was not represented. A total of 11 patients out of 17 were female (65%) and 6 were male (35%).

The most common cause of death could be attributed to the category *anesthesia, medication, or sedation* (14/17, 82%), followed by *bleeding or coagulation* (2/17, 12%) and *infection* (1/17, 6%). For a breakdown of the number of casualties due to *anesthesia, medication, or sedation* by age, see Multimedia Appendix 2.

Prior to their death, 13 out of 17 casualties (76%) had been treated under general anesthesia. In 1 case each out of 17 (6%), sedation or local anesthesia had been administered, and in 2 cases (12%) no details were given.

#### Online Results for the United Kingdom

The mean age of the 14 deceased patients was 30.5 years (SD 22.1; median 28.5 years). The dominating age groups were 6-17 years and 31-55 years, with 4 cases out of 14 (29%) each, whereas 2 patients (14%) in the age group 0-5 years died. The age group 18-30 years was represented with 1 case out of 14 (7%). Of the 14 deceased, 3 (21%) were in the 56-70-year age group, and the over-70-year age group was not affected at all. Half of the deceased were male (7/14, 50%) and half were female (7/14, 50%).

In the United Kingdom, the cause of death was attributed to the *anesthesia, medication, or sedation* category in 9 of the 14 cases (64%), to *bleeding or coagulation* in 3 cases (21%), and to *infection* and *others* in 1 (7%) case each. In the *others* category, a case of a small girl who had been starving to death after a traumatic visit to the dentist was described. For a breakdown of the number of deaths due to *anesthesia, medication, or sedation* by age, see Multimedia Appendix 3.

General anesthesia had been administered in 6 of the 14 cases (43%), sedation in 1 case (7%), and local anesthesia in 5 cases (36%). In addition, 2 patients (14%) did not receive any anesthesia at all.

#### Online Results for the United States

The mean age of the deceased patients was 22.6 years (SD 21.6; median 17 years). More than every second deceased patient was a minor: 25 out of 96 (26%) were in the 0-5-year age group, 29 (30%) were in the 6-17-year age group, 18 (19%) were in the 18-30-year age group, and 12 (13%) were in the 31-55-year age group. In the age group 56-70 years, 7 patients (7%) died, and 5 (5%) of the deceased were older than 70 years. Out of 96 deceased patients, 54 (56%) were male and 42 (44%) were female.

In the United States, 61 of 96 casualties (64%) were assigned to the *anesthesia, medication, or sedation* category. Out of 96 cases, 51 (53%) were related to anesthesia (ie, general anesthesia, sedation, or local anesthesia) and the other 10 cases (10%) were attributed to medication given before or after treatment (eg, pain medication and antibiotics). For a breakdown of the number of deaths due to *anesthesia, medication, or sedation* by age, see Multimedia Appendix 4. A total of 15 out of 96 (16%) deaths were assigned to the *airway or respiratory system* category, 8 cases (8%) to the *cardiovascular system*, 9 (9%) to *infection*, and 2 (2%) to *others*, which included 1 that was defined as a “natural cause of death” and 1 (1%) that was attributed to an asthma attack. Out of 96 cases, 1 (1%) was assigned to the *bleeding or coagulation* category.

In 29 out of 96 cases (30%), no information about the administered anesthesia had been reported. Most of the casualties had undergone sedation (44/96, 46%), and 16 patients (17%) had been treated under general anesthesia. In 7 cases (7%), only local anesthesia had been used.

From the United States, papers including 11 cases that had received nitrous oxide had been published. In 10 of these cases, the cause of death was assigned to *anesthesia, medication, or sedation*; these are subdivided in Table 2.

**Table 2:** Dental practice casualties involving nitrous oxide assigned to the anesthesia, medication, or sedation category from online publications in the United States.

Sedative agents		Casualties (n=10), n (%)
**Nitrous oxide with local anesthesia**		
	Total	3 (30)
	Nitrous oxide reported to be causative	1 (10)
**Nitrous oxide with sedative drugs or general anesthesia**		
	Total	7 (70)
	Nitrous oxide reported to be causative	2 (20)

In the 11th case where nitrous oxide was used, the cause of death was assigned to the *airway or respiratory system* category. This case had also been reported in the PubMed literature; the little girl had died after having aspirated a cotton roll. In only 1 of the 11 cases (9%), an anesthesiologist had been on site, and in 1 case (9%) there was no information regarding an anesthesiologist standing by.

In Table 3, all fatalities under general anesthesia and sedation were listed regarding the presence of an anesthesiologist or anesthetist. In the cases without a supervising anesthesiologist, a second dentist or even the treating dentist himself or herself had acted as *anesthesiologist*. It is unclear whether the respective dentists had completed an advanced training course prior to the procedures.

**Table 3:** Fatalities in dental practices under general anesthesia and sedation with respect to the presence of an anesthesiologist or anesthetist, based on data from online publications in the United States.

Setting of fatalities	Anesthesiologist present, n (%)	Anesthetist present, n (%)	No anesthesiologist or anesthetist present, n (%)	No information, n (%)
Fatalities where general anesthesia or sedation were used (n=60)	6 (10)	1 (2)	33 (55)	20 (33)
Fatalities where general anesthesia or sedation were used and cause of death was assigned to *anesthesia, medication, or sedation* category (n=47)	6 (13)	1 (2)	26 (55)	14 (30)

#### Online Results for France

Only 1 casualty occurred in France. The deceased was a 7-year-old boy who had aspirated a protective cover for the x-ray film and had suffocated in the dental office. Consequently, the death was classified as *airway or respiratory system*. No anesthesia had been administered to the patient.

### PubMed Publications

#### PubMed Publications in Total

**Figure 2:**
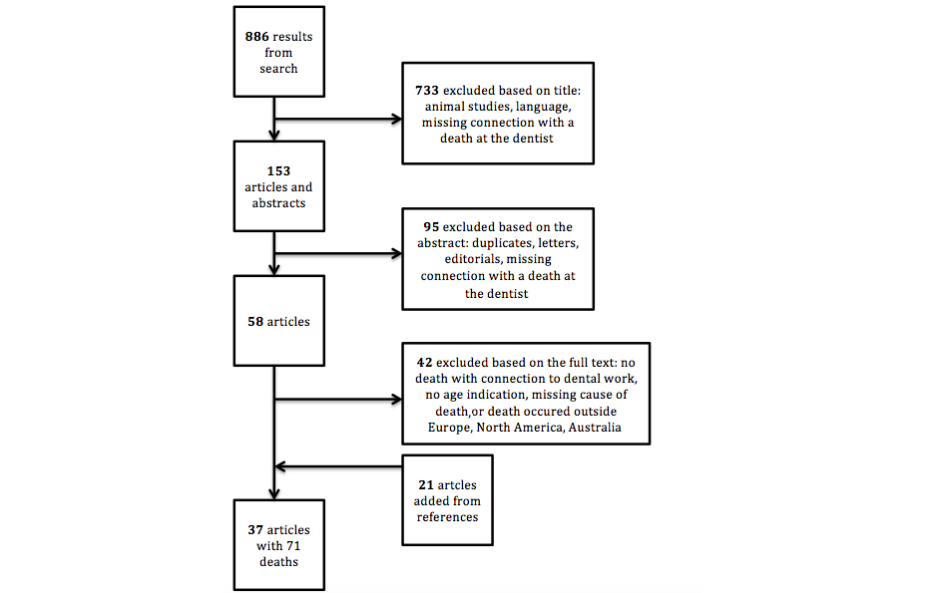
Flowchart for article selection from PubMed results.

We found 886 publications from our PubMed search. The flowchart in Figure 2 describes the process of exclusion and inclusion of publications. A total of 733 publications were excluded based on the title, 95 were excluded based on the abstract, and 58 publications underwent a full-text review. Finally, 37 publications reporting a total of 71 fatalities were included in the study. A total of 20 publications were case reports [9,10,12,13,18,20,22,24-26,28-37], 5 were retrospective studies [6,14,21], and 12 were case studies [7,8,11,17,19,23,26,38-42].

#### PubMed Results

The mean age of the 71 deceased from the PubMed literature was 37.9 years (SD 26.4; median 32 years) and included 43 (61%) male and 27 (38%) female patients. In 1 case (1%), the author had not specified a gender. The dominating age groups were 56-70 years and minors up to 5 years of age, with 15 cases (21%) each. The age group between 6 and 17 years was represented by 5 cases (7%), and the age group between 18 and 30 years was represented by 14 cases (20%). Of the 71 deceased, 12 (17%) died between 31 and 55 years of age and 10 (14%) were older than 70 years of age.

According to the PubMed literature, 25 cases out of 71 (35%) were assigned to the *anesthesia, medication, or sedation* category. A total of 24 cases (34%) were related to anesthesia (ie, general anesthesia, sedation, or local anesthesia), while the 25th patient died due to an anaphylactic shock originating from the dental impression material. The *infection* and the *cardiovascular system* categories were represented by 15 cases (21%) each, the *airway or respiratory system* category by 12 cases (17%), and the *bleeding or coagulation* category by 4 cases (6%). For a breakdown of the number of deaths due to *anesthesia, medication, or sedation* by age, see Multimedia Appendix 5.

The most common cause of death was from general anesthesia (22/71, 31%), followed by local anesthesia and sedation with 14 cases each (20%). Only 1 casualty (1%) had occurred completely without anesthesia. In the remaining 20 cases (28%), the author had not provided any information about the anesthesia administered.

Out of 71 fatalities, the 36 cases (51%) where general anesthesia or sedation had been used were examined with respect to the presence of an anesthesiologist or anesthetist (see Table 4).

**Table 4:** Fatalities in dental practices with general anesthesia and sedation with respect to the presence of an anesthesiologist or anesthetist, based on data from PubMed publications.

Setting of fatalities	Anesthesiologist present, n (%)	Anesthetist present, n (%)	No anesthesiologist or anesthetist present, n (%)	No information, n (%)
Fatalities where general anesthesia or sedation were used (n=36)	3 (8)	6 (17)	5 (14)	22 (61)
Fatalities where general anesthesia or sedation were used and cause of death was assigned to *anesthesia, medication, or sedation* category (n=19)	3 (16)	2 (10)	4 (21)	10 (53)

In PubMed, 11 fatalities where nitrous oxide had been used were described. In 8 of these cases (73%), the cause of death was classified as *anesthesia, medication, or sedation*. However, it was not obvious that nitrous oxide was the cause of death in any of the cases; only the involvement of nitrous oxide was mentioned. The other 3 cases (27%) involving nitrous oxide were assigned to *the airway or respiratory system* category. Those patients had died because of aspiration of a cotton roll, bronchospasm, or laryngeal edema. In Table 5, the PubMed data are compared with online results.

**Table 5:** Age and sex of casualties, cause of death, and anesthesia used in dental facilities found in online publications from three country groups compared with the PubMed literature.

Variable		Germany and Austria (n=17), n (%)		United Kingdom (n=14), n (%)		United States (n=96), n (%)		PubMed (N=71), n (%)	
**Age (years)**									
	0-5		8 (47)		2 (14)		25 (26)		15 (21)
	6-17		2 (12)		4 (29)		29 (30)		5 (7)
	18-30		3 (18)		1 (7)		18 (19)		14 (20)
	31-55		3 (18)		4 (29)		12 (13)		12 (17)
	56-70		0 (0)		3 (21)		7 (7)		15 (21)
	>70		1 (6)		0 (0)		5 (5)		10 (14)
**Sex**									
	Female		11 (65)		7 (50)		42 (44)		27 (38)
	Male		6 (35)		7 (50)		54 (56)		43 (61)
	Not specified		0 (0)		0 (0)		0 (0)		1 (1)
**Cause of death**									
	Anesthesia, medication, or sedation		14 (82)		9 (64)		61 (64)		25 (35)
	Bleeding or coagulation		2 (12)		3 (21)		1 (1)		4 (6)
	Infection		1 (6)		1 (7)		9 (9)		15 (21)
	Others		0 (0)		1 (7)		2 (2)		0 (0)
	Cardiovascular system		0 (0)		0 (0)		8 (8)		15 (21)
	Airway or respiratory system		0 (0)		0 (0)		15 (16)		12 (17)
**Anesthesia**									
	General anesthesia		13 (76)		6 (43)		16 (17)		22 (31)
	Sedation		1 (6)		1 (7)		44 (46)		14 (20)
	Local anesthesia		1 (6)		5 (36)		7 (7)		14 (20)
	None		0 (0)		2 (14)		0 (0)		1 (1)
	No information		2 (12)		0 (0)		29 (30)		20 (28)

### Comparison of PubMed Literature With Summarized Online Publications

#### Age

The mean age of the deceased patients in online publications was 22.9 years (SD 21.6; median 17 years) compared with 37.9 years (SD 26.4; median 32 years) in PubMed articles. For a compilation of the age distribution, see Multimedia Appendix 6.

#### Cause of Death

**Figure 3:**
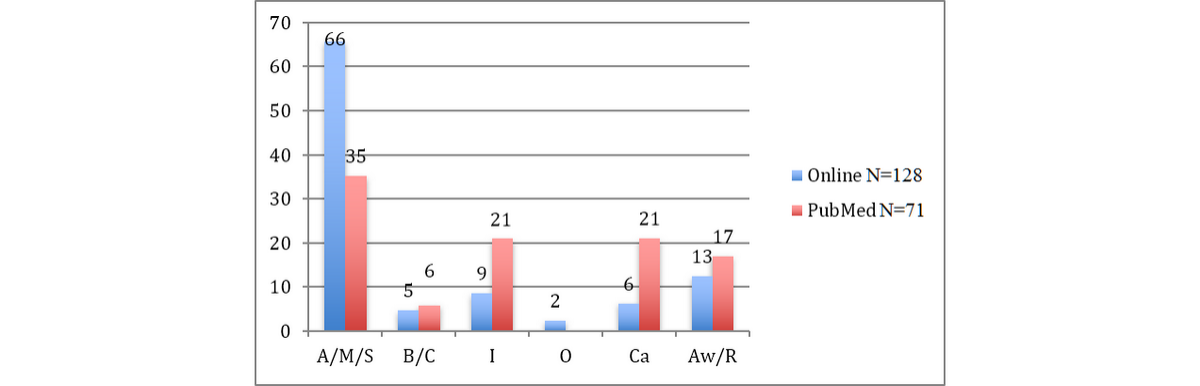
Percentage of casualties for each cause of death in dental facilities using data from online publications and PubMed. Possible duplicates were not excluded. A/M/S: anesthesia, medication, or sedation; B/C: bleeding or coagulation; I: infection; O: others; Ca: cardiovascular system; Aw/R: airway or respiratory system.

The *anesthesia, medication, or sedation* category dominated the online and the PubMed publications (see Table 5); however, the proportions in PubMed were only about half as high compared to online publications. Whereas *bleeding or coagulation* problems did not show a large difference, *infection*, *cardiovascular system*, and *airway or respiratory system* problems were overly represented in PubMed (see Figure 3).

#### Anesthesia

Whereas local anesthesia as the reason for fatal incidents showed a higher proportion in PubMed publications, sedation was more strongly represented in online reports. No large differences in general anesthesia fatality rates were found. For a breakdown of the types of anesthesia used, see Multimedia Appendix 7.

#### Anesthesia and Age

**Figure 4:**
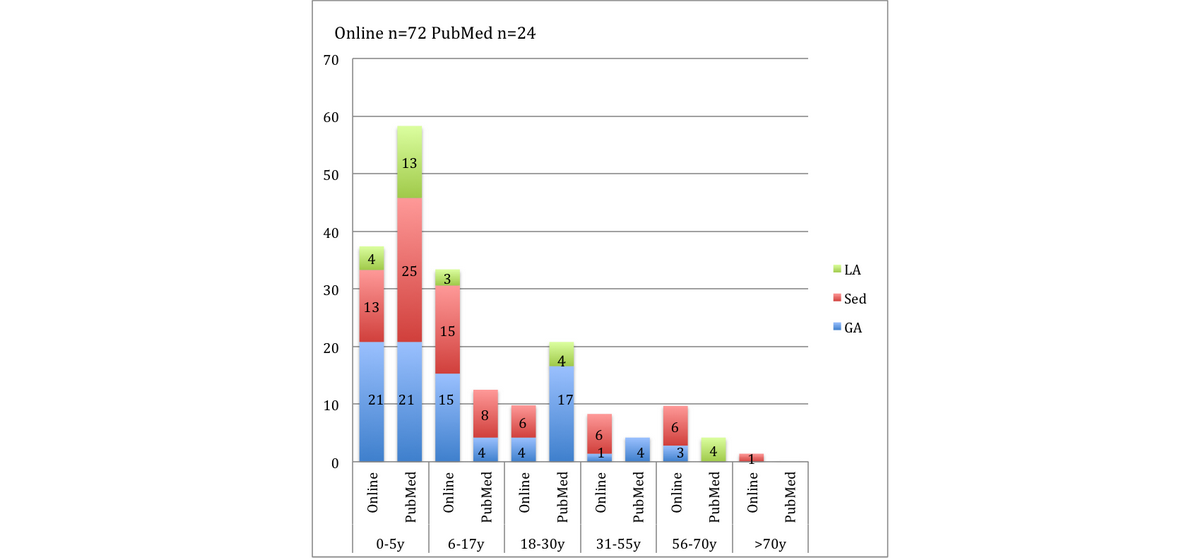
Percentage of deaths in dental facilities reported in online and PubMed publications categorized as *anesthesia, medication, or sedation* with the use of general anesthesia (GA), sedation (Sed), or local anesthesia (LA) in each age group (years, y). Possible duplicates were not excluded.

The analysis of PubMed articles showed higher proportions of both sedation and local anesthesia leading to fatalities among patients 5 years of age and younger compared to online publications. However, the proportion of sedation leading to fatalities dominated the 6-17-year-old and 31-years-and-older age groups in online media. General anesthesia showed higher percentages in online publications among the 6-17-year-old and 56-70-year-old age groups and higher percentages in PubMed articles among the 18-30-year-old and 31-55-year-old age groups (see Figure 4).

### Comparison by Chi-Square Statistics

#### Selection of Publications to Compare

Out of 128 deaths found in online publications, 11 cases (8.6%) were excluded for statistical analysis, as these were also found in the PubMed literature. Therefore, 117 cases from online publications were compared with 71 cases from the PubMed literature using chi-square statistics. When comparing the three country groups—Germany and Austria, the United Kingdom, and the United States—all cases were included.

#### Age

A higher proportion of fatalities in the 17-years-and-younger age group was found in online publications (68/117, 58.1%) compared to PubMed articles (20/71, 28%). However, among the group of patients older than 70 years, fatal outcomes were found in 14% (10/71) of PubMed publications compared to 4.3% (5/117) of online publications (see Table 6).

**Table 6:** Comparison of fatalities reported in online and PubMed publications with respect to the age of deceased patients.

Patient age group		Number of fatalities, n (%)		*P* value
		Online (N=117)	PubMed (N=71)	
**Over and under 18 years**				<.001
	<18 years	68 (58.1)	20 (28)	
	≥18 years	49 (41.9)	51 (72)	
**Over and under 70 years**				.02
	≤70 years	112 (95.7)	61 (86)	
	>70 years	5 (4.3)	10 (14)	

#### Causes of Death

Fatalities in the *anesthesia, medication, or sedation* category dominated online, whereas *infection* and *cardiovascular system* rates were higher in PubMed. No differences between online and PubMed publications were found when *airway or respiratory system* and *bleeding or coagulation* categories were compared (see Table 7).

**Table 7:** Comparison of fatalities reported in online and PubMed publications with respect to causes of death.

Cause of death		Number of fatalities, n (%)		*P* value
		Online (N=117)	PubMed (N=71)	
**Anesthesia, medication, or sedation**				<.001
	Yes	80 (68.4)	25 (35)	
	No	37 (31.6)	46 (65)	
**Infection**				.01
	Yes	10 (8.5)	15 (21)	
	No	107 (91.5)	56 (79)	
**Cardiovascular system**				<.001
	Yes	5 (4.3)	15 (21)	
	No	112 (95.7)	56 (79)	
**Airway or respiratory system**				.26
	Yes	13 (11.1)	12 (17)	
	No	104 (88.9)	59 (83)	
**Bleeding or coagulation**				.88
	Yes	6 (5.1)	4 (6)	
	No	111 (94.9)	67 (94)	

### Comparison Regarding the Use of Sedation

The number of fatalities attributed to sedation were higher in online reports covering the US population compared to data from other countries (see Table 8). The proportion of sedation as the reason for fatal outcomes was higher in the online reports (41/117, 35.0%) compared to articles from PubMed (14/71, 20%) (see Table 9).

**Table 8:** Comparison of fatalities in dental facilities as a result of sedation reported in online publications with respect to country.

Country	Fatalities resulting from sedation, n (%)	Fatalities not resulting from sedation, n (%)	*P* value
Germany and Austria (n=17)	1 (6)	16 (94)	N/A^a^
United Kingdom (n=14)	1 (7)	13 (93)	N/A
United States (n=96)	44 (46)	52 (54)	N/A
Germany and Austria vs United Kingdom	N/A	N/A	.89
Germany and Austria vs United States	N/A	N/A	.002
United Kingdom vs United States	N/A	N/A	.006

^a^N/A: not applicable.

**Table 9:** Comparison of fatalities in dental facilities as a result of sedation in online and PubMed publications.

Publication source	Fatalities resulting from sedation, n (%)	Fatalities not resulting from sedation, n (%)	*P* value
Online (N=117)	41 (35.0)	76 (65.0)	.03
PubMed (N=71)	14 (20)	57 (80)	

### Comparison Before 1995 and After 1994

To fully exclude duplicates, the online cases after 1994 were compared with the PubMed cases before 1995. As shown in Table 10, there is no difference between the respective time frames regarding PubMed or when the numbers in the categories leading to fatalities were compared.

Whereas data identifying *anesthesia, medication, or sedation* as contributing factors dominated in online reports after 1994 compared to publications listed in PubMed before 1995, *sedation*-related casualty proportions in both of the examined databases were not different (see Table 11).

**Table 10:** Comparison of fatalities in dental facilities described in PubMed before 1995 and after 1994 with respect to deaths assigned to the anesthesia, medication, or sedation and sedation-only categories.

Cause of death		Fatalities reported in PubMed, n (%)			*P* value		
		Before 1995 (n=23)		After 1994 (n=48)			
**Anesthesia, medication, or sedation**						.63	
	Yes		9 (39)	16 (33)			
	No		14 (61)	32 (67)			
**Sedation only**						.77	
	Yes		5 (22)	9 (19)			
	No		18 (78)	39 (81)			

**Table 11:** Comparison of fatalities in dental facilities described in PubMed before 1995 and online after 1994 with respect to deaths assigned to the anesthesia, medication, or sedation and sedation-only categories.

Cause of death		Fatalities reported, n (%)		*P* value
		PubMed before 1995 (n=23)	Online after 1994 (n=108)	
**Anesthesia, medication, or sedation**				.008
	Yes	9 (39)	74 (68.5)	
	No	14 (61)	34 (31.5)	
**Sedation only**				.24
	Yes	5 (22)	37 (34.3)	
	No	18 (78)	71 (65.7)	

## Discussion

### Principal Findings and Implications

#### Overview

The main finding of this study was that reports regarding causes of death in dental offices published on the internet enhance knowledge about the respective fatalities compared to extracting data from PubMed only. Fatalities associated with medication, general anesthesia, and sedation would have been underrepresented if only examining fatalities published in PubMed without considering cases reported on the internet. The same applies to the fatality rates of minors having died related to a dental treatment. In comparison to Reuter et al [4], the PubMed literature review conducted in this study was restricted to the last 30 years and to publications from Europe, North America, and Australia. Therefore, a lower number of death cases were obtained in this study’s PubMed search. This procedure was applied in order to use the same criteria in both the PubMed and online searches and to enable a reasonable comparison of the results. Reuter et al’s publication [4], as well as this study, revealed a fatality value of 21% for the category *cardiovascular system*; in addition, as a main result, it can be concluded that in both works most fatalities were assigned to the category *anesthesia, medication, or sedation*.

#### Age

The differing mean age in online publications (mean 22.9 years, SD 21.6; median 17 years) and in the PubMed literature (mean 37.9 years, SD 26.4; median 32 years) can possibly be explained by an increased awareness by the public when younger people die as a result of dental treatment.

On the other hand, the authors of the PubMed articles evaluated available data using a scientific approach. This may explain the higher average age in scientific literature as reflecting a more even age distribution.

#### Cause of Death

##### Overview

Death categories in PubMed articles were more evenly distributed compared to online publications. In both the online publications and in the PubMed literature, the most common cause of death could be assigned to the *anesthesia, medication, or sedation* category; the percentage in online publications was 65.6% (84/128), which is almost twice as high as in the PubMed articles (25/71, 35%). 

Presumably, articles describing deaths associated with anesthesia or sedation (ie, *anesthesia, medication, or sedation* category) may induce a compassionate effect in a normal reader and are, therefore, more suitable for headlines, especially when the group of patients is under 18 years of age, which was overly represented.

Furthermore, it is noticeable that only 11 out of 128 (8.6%) of the fatalities reported in online publications occurred after infection, whereas 21% (15/71) of infection-related fatalities were found in PubMed articles. This could be explained by symptoms of infection having developed with delay after dental treatment. Compared with a death directly related to the administration of anesthesia or sedation for dental treatment, delayed infection may be less suitable as an online headline.

In addition, a clear difference between online publications (8/128, 6.0%) and PubMed articles (15/71, 21%) in the category *cardiovascular system* was found. This difference may have resulted from the higher average age of the patients in the PubMed articles. Furthermore, it is assumed that deaths in the *cardiovascular system* category were not associated with dental treatments, especially by medical laypersons.

##### Anesthesia, Medication, or Sedation 

In the case of deaths attributed to the category *anesthesia, medication, or sedation*, omissions in the use of adequate equipment or medication overdose had predominantly been responsible for the death of the patient. Furthermore, medication side effects or undiscovered diseases had sometimes been attributed to the fatal outcome. Regarding these causes of death, dentists’ adequate knowledge of the equipment, medication, and respective complications is rated as a major issue. Whether anesthesiologists have been underinformed or have been ignoring related health or safety issues in the respective cases remains unclear.

Data from the 1960s until now already show an impact of anesthesia, medication, or sedation procedures on fatalities in dental facilities. However, some bias may arise regarding substances historically available that are no longer in use in many countries, such as halothane [4]. Enhanced knowledge about fatalities in dental facilities covering the last 30 years has been obtained from additional information derived from internet reports compared to PubMed literature alone within this time frame. Although some historical medication identified as a cause of death in previous publications is no longer available, performing sedation or even general anesthesia in certain dental facilities without the assistance of an anesthesiologist is still considered dangerous.

Frequently, online publication analysis revealed that both dentists and dental staff had had no or insufficient knowledge of lifesaving procedural management. Regular emergency management and cardiopulmonary resuscitation training is advisable [43]. Emergency management of the respective complications would not be necessary if at-risk interventions, such as deep sedation or even general anesthesia, by dentists could be avoided, since these complications would not even occur. Dentists should probably be discouraged from performing these procedures. Awareness of the actual status of these procedures in the dental office as well as knowledge of the definition of *deep sedation* and *general anesthesia* may be of major concern.

##### Bleeding or Coagulation

Deaths attributed to the category *bleeding or coagulation* may have occurred because of underestimation of the bleeding risk. Although a coagulation disorder or the use of anticoagulants was known, surgical procedures had often been performed without consulting the patient’s physician or without using adequate hemostyptic medical devices. In online publications, cases had also been reported in which patients had not returned to the dental office despite recurrent bleeding and had then bled to death, for example, during sleep. It is quite possible that patients may underestimate a rebleeding episode and, therefore, avoid another consultation with the dentist. In particular, because of an increasing number of patients taking anticoagulant medication, the importance of an accurate survey of the patient’s history and consultation with the patient’s physician, as well as sufficient instructions for the patient after a surgical procedure, is highlighted [44]. Furthermore, an accurate assessment of a patient’s bleeding risk enables the dentist to be prepared for intra- or postoperative bleeding (eg, with topical hemostatic agents or mechanical techniques) [45].

Other causes of death in this category are unknown diseases, for example, liver-related diseases, which may also influence the coagulation system. Unfortunately, these risks cannot be completely ruled out, even if the patient is carefully examined and the surgery performed properly.

##### Infection

In the PubMed literature, in particular, a considerable number of deaths that were categorized as *infection* and deaths because of endocarditis were described. While 7 cases were found in PubMed, only 1 case was reported in online publications, where a patient had died because of endocarditis. In 4 of these cases, it was stated that the treating dentist had not administered antibiotic prophylaxis before the treatment. In the other 3 cases, this information could not be clearly tracked from the publication. With an incidence of 3-7 cases per 100,000 people, infectious endocarditis is a rare but serious disease with a mortality rate of 20%-25% [46-50].

Dentists need to be aware of the indications for administering antibiotic prophylaxis. The recommendations to administer antibiotic prophylaxis to patients at risk before dental treatment have recently undergone certain modifications [51]. To prevent infectious endocarditis, dentists should consult the patient’s physician or cardiologist when treatment of at-risk patients is planned.

##### Cardiovascular System

In both the online publications and the PubMed literature, deaths assigned to the *cardiovascular system* category were described. As reported earlier [4], in this study, cases were occasionally found in which a relationship to the anesthesia or medication used could not be completely ruled out. Nevertheless, as in other categories, a detailed survey of the patient’s history is essential and, if necessary, a consultation with the patient’s physician or cardiologist would be recommended. A fatal cardiovascular incident can occur anywhere; in a dental office, a distinction is made between cases that would have occurred independently from dental treatment and cases triggered by the treatment (eg, stress and local anesthesia) [43]. Air embolism is a very rare complication but has been described during drilling procedures for dental implant placement. Therefore, air-operated handpieces are obsolete for this procedure, and the equipment recommended by the manufacturer of the implants should be used [52].

##### Airway or Respiratory System

In online publications, about half of the deaths in the *airway or respiratory system* category had been caused by aspiration of foreign bodies (eg, tooth, cotton roll, swabs, and instruments) or stomach contents. However, swelling (eg, abscess and hematoma) with airway occlusion, as well as 1 case of suffocation having resulted from the patient’s position during treatment, has been described as well. In the PubMed publications, mainly hereditary angioedema had been responsible for the described fatalities. As it can be triggered even by oral manipulation during dental treatment, a patient’s lack of knowledge even after having been informed about the diagnosis years ago may have contributed to the occurrence. Since the onset of swelling may be delayed more than 24 hours after a dental procedure, the dentist may not even be involved in emergency management. Therefore, a survey of the patient’s history should involve questions about these respective issues. In cases with a positive history, dental treatment should only be performed after consultation with the primary care physician [23,53].

Aspiration of foreign bodies may be prevented using protective membranes, such as rubber dams, especially in endodontic treatments [54]. However, this does not protect against aspiration of extracted teeth, cotton rolls, or gauze positioned in the throat in cases of general anesthesia and endotracheal intubation.

#### Anesthesia and Sedation

##### Overview

Anesthesia and sedation are considered priority topics in both this and other studies [4], therefore, they are discussed in more detail in this section. While the proportions of general anesthesia related to the respective deaths were almost equal in online publications (35/128, 27.3%) and in the PubMed literature (22/71, 31%), differences were noted in the use of sedation and local anesthetics. Under sedation, the percentage of deaths in online publications (46/128, 35.9%) was almost twice as high as those in the PubMed articles (14/71, 20%). Compared with general anesthesia, sedation of dental patients might be perceived as a harmless procedure.

The primary concern of the authors of online publications had probably not been to draw attention to the underestimation of risks by dental sedation. However, the additional knowledge extracted from the internet reports in this study may contribute significantly to the awareness of these risks in the dental community.

##### Local Anesthesia

In online publications, 10.2% (13/128) of the described deaths had been attributed to local anesthetic treatment compared with 20% (14/71) in the PubMed literature. It is unclear whether the deaths caused by this type of anesthesia are underreported on the internet.

The importance for including cases with supposedly less-harmful local anesthetic for the professional world is emphasized to draw attention to the risks and to define measures of care. It is noticeable that fatalities under local anesthesia in the PubMed articles covered mainly the group of children up to 5 years of age, where the dentists may have underestimated the risk of overdose.

##### Sedation and General Anesthesia

In online publications and in the PubMed literature, cases were described in which nitrous oxide had been used. In the specialist literature, however, no direct involvement of nitrous oxide in the death of the patients could be detected. In the fatalities described in online publications from the United States, nitrous oxide had been reported to be causative in 3 out of 10 cases (30%). A limitation could be the perception of the author of the respective internet post, who may have reported speculation as fact. In addition, a coroner’s report had not been cited.

Nitrous oxide is still widely used in dentistry. In online publications, deaths involving nitrous oxide are reported exclusively from the United States. This may be due to the perception of maximum safety of nitrous oxide. In the last 30 years, the number of states where no further approval is needed for its administration has been decreasing [55].

Only 1 sedation case could be retrieved online from Germany and Austria. Younger fatalities, especially below 5 years of age, had occurred under general anesthesia.

As there were no respective fatality reports from online publications covering the United Kingdom, the German-speaking countries, as well as France, nitrous oxide involvement can only be analyzed from the US data. Nitrous oxide had contributed to the fatal course of treatment in only 10 cases. Because of the low number, no statement can be made about whether additional sedative or anesthetic drugs involved may have enhanced the medical problem leading to death. Only 1 fatality caused by foreign body aspiration in connection to nitrous oxide was published on the internet. However, as extracted from the PubMed database, the problem of aspiration and related issues, such as bronchospasm and laryngeal edema, seems to be more relevant during nitrous oxide use. This should increase the perception of the respective risk under adjunctive nitrous oxide or additional sedation during dental treatment under local anesthesia. It is expected that in most situations where nitrous oxide is used in dental treatment facilities the dentist has no additional anesthesiologist support. However, in the data obtained here, additional sedative medication had been used in 7 fatalities and in only 1 case an anesthesiologist was standing by.

In Germany and Austria, the predominant adjunctive measures had been general anesthesia performed by a certified anesthesiologist, whereas sedation procedures or even general anesthesia had been performed without a certified anesthesiologist in a significant number of cases in the United States. Sedation-related problems leading to the death of a patient seem to be more relevant in the United States compared with the other areas examined (see Table 8).

A significant association of deaths due to anesthesia or sedation with the presence or absence of an anesthesiologist has been demonstrated [4]. In more than 50% of fatalities reported in online publications covering the United States, where adjunctive general anesthesia or sedation had been used, it became obvious that no anesthesiologist had been on site during either general anesthesia or sedation. Our PubMed search found only 5 cases out of 36 (14%) where no anesthesiologist had been on site during adjunctive general anesthesia or sedation procedures. However, in 22 out of 36 cases (61%), no information about a specialist standing by had been provided. Nevertheless, this difference is remarkable, as the internet reports regarding this category almost exclusively cover the United States database. Even if an overlap between the internet and PubMed databases cannot fully be excluded, the high percentage in the internet reports may increase awareness among clinicians that the factor *anesthesiologist on site* is significantly influencing the course of respective incidents. This factor would have been underreported without the internet analysis.

It is also remarkable that in more than one-third of deaths reported in the PubMed literature and in almost two-thirds of deaths found in the internet publications covering the United States and the United Kingdom, an *anesthesia, medication, or sedation*-related problem was at least partly involved in the fatal outcome (see Table 5). Data may focus the awareness of dental practitioners—not only those in the United States—on anesthesiologist support, especially when sedative medication is used. Thereby, patient safety would be enhanced and preventable deaths of patients at risk may significantly be reduced.

PubMed data have not shown an increase of fatalities where sedation, as such, was involved when publications from the era before internet data availability were compared with those from the internet age beginning around 1995. Furthermore, when comparing the internet data after the beginning of 1995 (ie, after 1994) with PubMed data before 1995, no differences can be found regarding sedation-associated fatality rates in relation to fatality rates not associated with sedation. However, a much higher proportion of fatalities in the *anesthesia, medication, or sedation* category can be extracted from the internet reports. This category would have been underestimated if the internet reports had not been included in this study.

In 2001, a recommendation from the Department of Health in the United Kingdom was published and restricted general anesthesia for dental treatment to the hospital setting [56].

### Limitations and Weaknesses of the Applied Method

First, a standardized evaluation is difficult since some of the online reports were written by laypersons without scientific backgrounds. The authors may have reported incorrect medical data or may have used incorrect terminology, possibly resulting in the assignment of a death to an inappropriate category. Second, the assignment of the cases from the internet and PubMed publications to the cause of death categories was difficult and was based on the authors’ impressions. Some fatalities could have been allocated to more than one category. Third, terms that referred to deaths in dental offices were used, but there might be other terms that could be related to dental deaths that were not searched for when browsing the internet. Despite a precise search using Google, there might be cases that may not have been taken into account. Fourth, care was taken to exclude possible duplicate cases. By comparing fatalities before 1995 and after 1994, we have tried to fully separate the populations examined. Nevertheless, duplicates cannot be ruled out completely. Fifth, the search was restricted to six countries, which might limit the generalizability. Further research might be needed to identify differences or overlaps in dental deaths in other countries.

### The Internet as a Data Source Evaluating Casualties in Dental Facilities

“Death in the dental office” may be a phrase that comes into the view of a regular online visitor, for example, on a news page. However, it is not a term of interest for the general public. The number of internet searchers was so low that Google Trends database analysis has not created statistics since 2004. However, dental clinicians or researchers may benefit to a large extent from the data obtained from such searches.

The implication of scientific publications indexed by PubMed may be significantly enhanced by adding data obtained from Google database searches, such as the topic in this paper.

### Conclusions

In this study, deaths having occurred in dental offices published on the internet and in PubMed were examined. It is advisable to be aware of the risks, no matter how low; the risk factors; and the diseases associated with dental offices in order to prevent possibly life-threatening situations in relation to dental treatment. The additional searches on the internet revealed relevant data regarding fatalities, which would have remained hidden if only extracting data from PubMed. Fatalities associated with general anesthesia or sedation would have been underrepresented, as well as the number of minors having died related to dental treatments, without the analysis of internet-based reports.

Regarding anesthesia, especially when used with children and even with local anesthetics, the risk of overdose should not be underestimated. The type and dose of medication should be of major concern. The definitions and understanding of deep sedation and general anesthesia should be a focus in the education of dentists willing to perform adjunctive sedation during treatment, in order to be aware of associated life-threatening problems. By avoiding potentially hazardous procedures, such as sedation-aided treatments performed exclusively by the dentist without the assistance of an experienced physician or anesthesiologist, the risk of treatment-induced life-threatening emergencies may be reduced.

## Appendix

Multimedia Appendix 1 Searches for the words "death" and "dental" in the United States using Google Trends.

Multimedia Appendix 2 Number of deaths due to *anesthesia, medication, or sedation* by age group using data from online publications in Germany and Austria.

Multimedia Appendix 3 Number of deaths due to *anesthesia, medication, or sedation* in each age group using data from online publications in the United Kingdom.

Multimedia Appendix 4 Number of deaths due to *anesthesia, medication, or sedation* in each age group using data from online publications in the United States.

Multimedia Appendix 5 Number of deaths in the *anesthesia, medication, or sedation* category due to general anesthesia, sedation, or local anesthesia in each age group from the PubMed literature.

Multimedia Appendix 6 Percentage of casualties in dental facilities for each patient age group, based on data from online publications and PubMed. Possible duplicates were not excluded.

Multimedia Appendix 7 Percentage of anesthesia types used in cases of casualties in dental facilities, based on data from online publications and PubMed.
