# Supplementary figures and images for: Using Nonexpert Online Reports to Enhance Expert Knowledge About Causes of Death in Dental Offices Reported in Scientific Publications: Qualitative and Quantitative Content Analysis and Search Engine Analysis
Source: J Med Internet Res. 2020 Apr 17;22(4):e15304. doi: 10.2196/15304 (PMC7195661; doi:10.2196/15304)

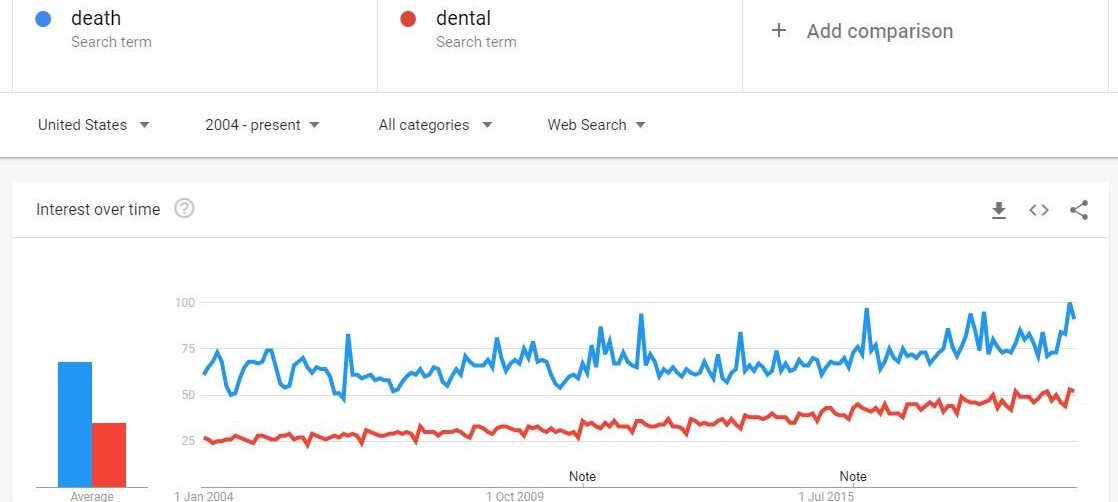

Supplement: Multimedia Appendix 1 [file jmir_v22i4e15304_app1.png]

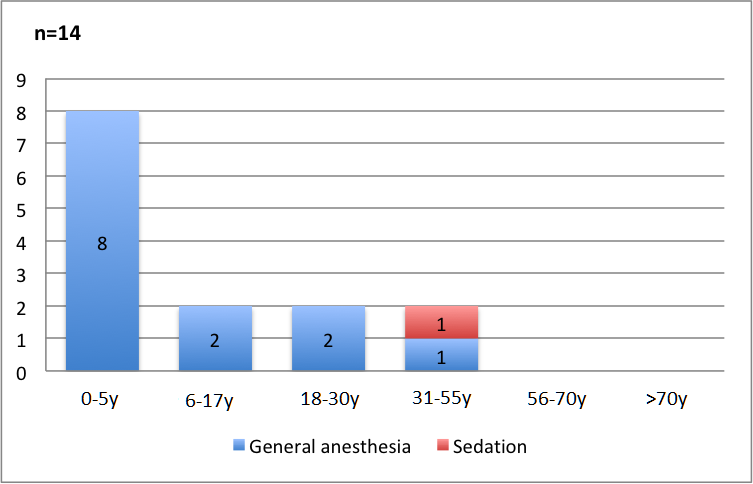

Supplement: Multimedia Appendix 2 [file jmir_v22i4e15304_app2.png]

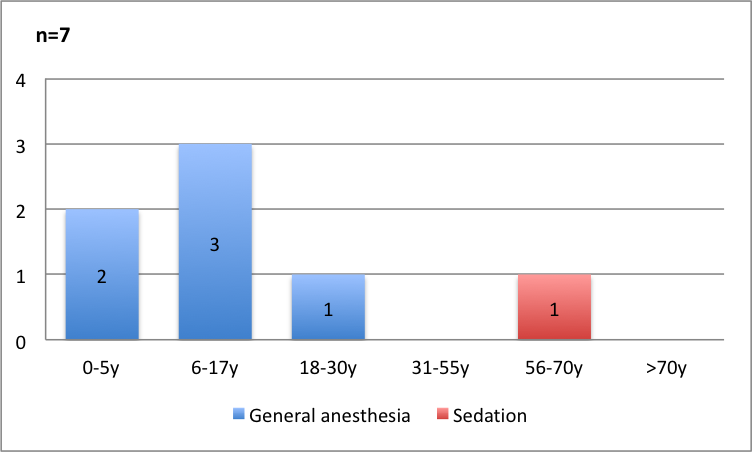

Supplement: Multimedia Appendix 3 [file jmir_v22i4e15304_app3.png]

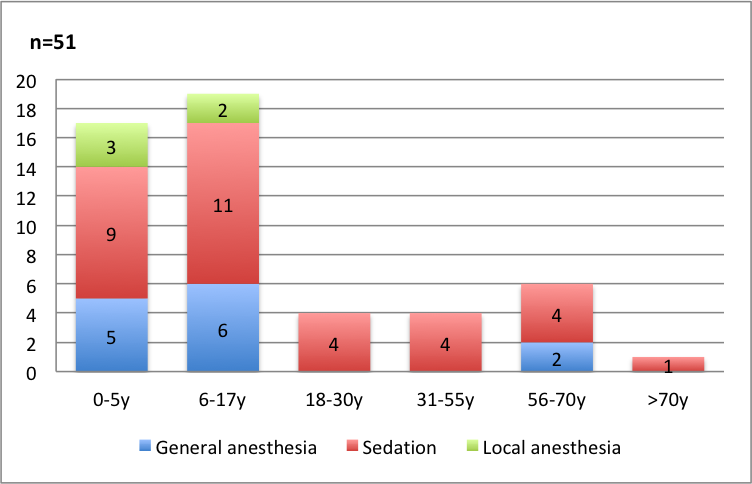

Supplement: Multimedia Appendix 4 [file jmir_v22i4e15304_app4.png]

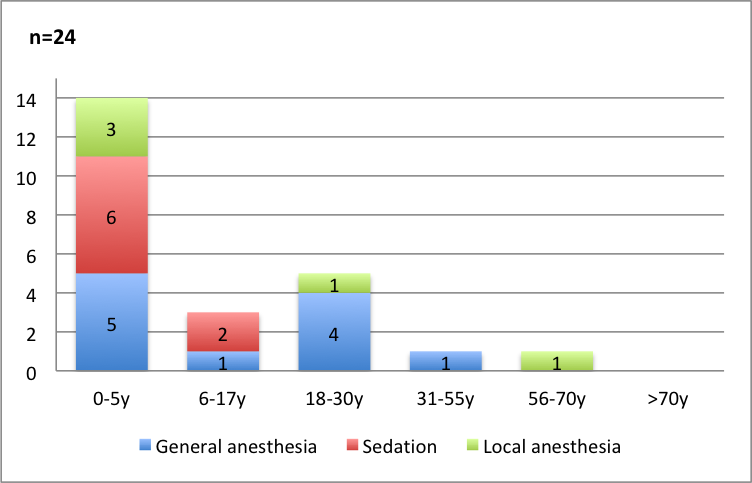

Supplement: Multimedia Appendix 5 [file jmir_v22i4e15304_app5.png]

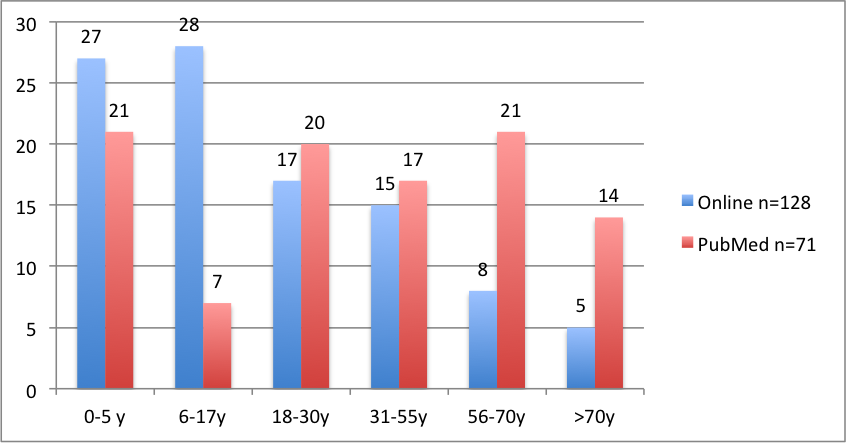

Supplement: Multimedia Appendix 6 [file jmir_v22i4e15304_app6.png]

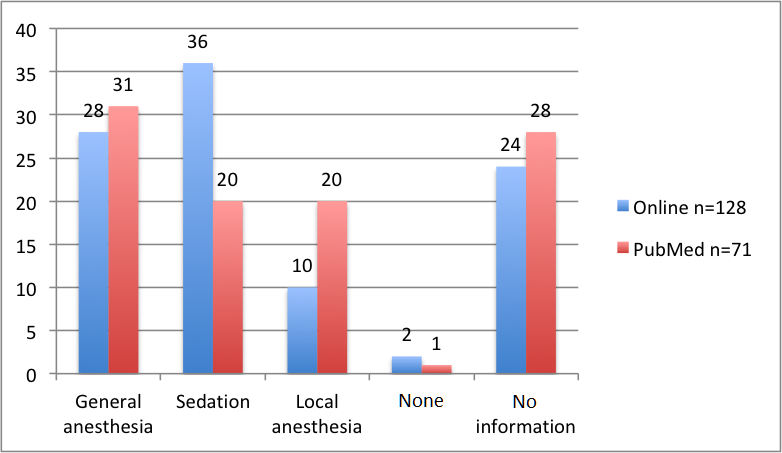

Supplement: Multimedia Appendix 7 [file jmir_v22i4e15304_app7.png]
